# Systematic Review and Meta-analysis of the Survival Outcomes of First-line Treatment Options in High-risk Prostate Cancer

**DOI:** 10.1038/srep07713

**Published:** 2015-01-12

**Authors:** Jun H. Lei, Liang R. Liu, Qiang Wei, Shi B. Yan, Tu R. Song, Fu S. Lin, Lu Yang, De H. Cao, Hai C. Yuan, Wen B. Xue, Xiao Lv, Ying C. Cai, Hao Zeng, Ping Han

**Affiliations:** 1 Department of Urology, West China Hospital, Sichuan University, Chengdu, China; 2 Department of Urology, Dujiangyan Medical Center/ the affiliated hospital of Chengdu University, Dujianyan, China

## Abstract

Prostate cancer (PCa) is the most common non-dermatologic cancer in the western countries in western countries. High-risk PCa accounts for 15% of the diagnosed cases. In this study, we compare the long-term survival outcomes of radical prostatectomy (RP), radiation therapy (RT), brachytherapy (BT), androgen- deprivation therapy (ADT), and watchful waiting (WW) in high-risk prostate cancer (PCa). Overall, RP/(RT plus ADT) gave the best survival outcome in patients with high-risk PCa, whereas ADT/WW had the worst outcome. The overall priority for treatment strategy could be ranked as follows: RP/(RT plus ADT), RT, and ADT/WW. RP had significant better overall survival (OS) than RT or BT, and RP had significant lower cancer-specific mortality (CSM) than RT (0.51 [95% CI 0.30–0.73], P<0.001). ADT improved the cancer-specific survival (CSS) of RP based on a case-controlled study; added ADT to RT failed to challenge the position of RP but could improve the outcome of RT. In conclusions,RP/(RT plus adjuvant ADT) could both be used for the first-line therapy of high-risk PCa. When encountering an individual patient, urologists should consider various factors like tumors themselves, preferences of individuals, and so on.

Prostate cancer (PCa) is the most common non-dermatologic cancer in the western countries in western countries. Epidemiological data show that its morbidity is approximately 0.214% in males and 192,000 individuals are diagnosed with PCa annually in the United States^1^.High-risk PCa accounts for 15% of the diagnosed cases^2^.The common treatment options for high-risk PCa are radical prostatectomy (RP), radiation therapy (RT), brachytherapy (BT), and androgen-deprivation therapy (ADT)^3^.

Although a consensus has not been reached for the definition of high-risk PCa, the D'Amico classification system is currently widely used for risk stratification in PCa. It comprises a three-point scale for recurrence and metastasis: low risk, medium risk, and high risk. High-risk PCa is defined as a prostate-specific antigen (PSA) level > 20 ng/ml, a Gleason score of 8–10, or a clinical stage ≥ T2c according to the American Urological Association (AUA) guideline^4^,^5^. However, the definition in the guidelines of the European Association of Urology (EAU) and the National Comprehensive Cancer Network (NCCN) is PSA>20 ng/ml, Gleason score 8–10, or clinical stage ≥T3a^6^. In addition, the Radiation Therapy Oncology Group (RTOG) also defined high-risk PCa as PSA 20-100 ng/ml, Gleason score 8–10, and any clinical stage of pT or PSA<100 ng/ml, Gleason score 8–10, and clinical stage ≥T2c^7^.

Because high-risk PCa is prone to recurrence and metastasis after treatment, an increasing number of studies have focused on this issue. Unfortunately, there is no consensus regarding the optimal treatment choice^8^. In the current study, we performed a systematic review of the literature to compare the long-term survival outcomes of RP, RT, BT, ADT, and watchful waiting (WW), alone or in combination, in patients with high-risk PCa.

## Results

### Study characteristics

**Figure 1:**
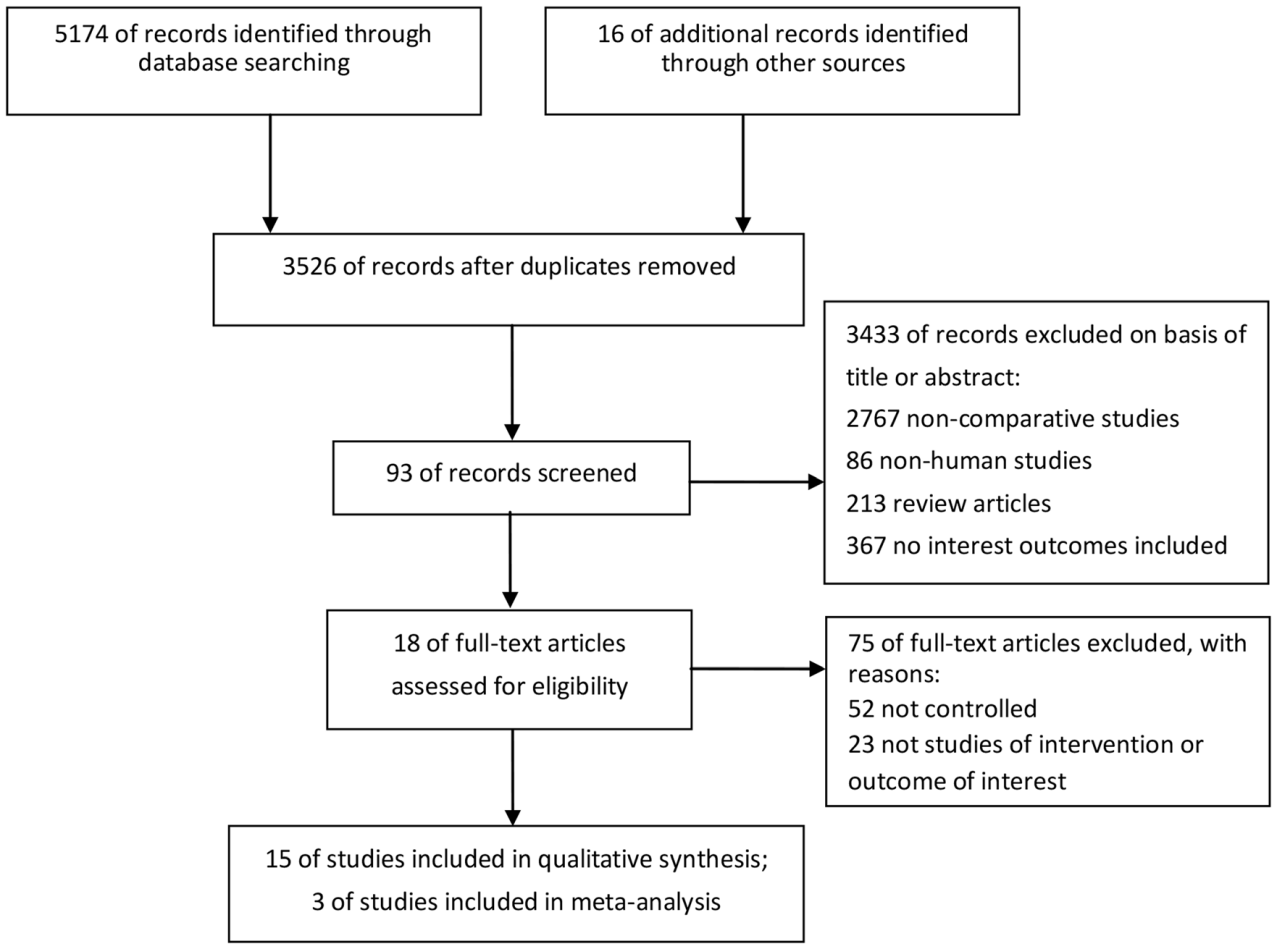
Flowchart of literature searches.

**Figure 2:**
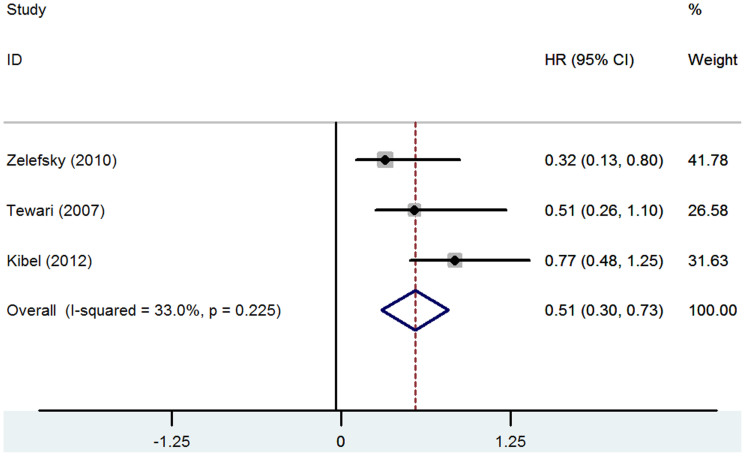
Forest plot of pooled hazard ratio (HR) for cancer-specific mortality (CSM).

A flowchart of the literature searches is shown in Figure 1. Of the 18 studies including 6986 patients, six compared different approaches without combined regimens (*N* = 3682) and the remaining 12 used combined regimens (*N* = 3304). The characteristics of the included studies are summarized in Table 1. Meta-analysis was performed only for the CSM of Zelefsky et al.^16^, Tewari et al.^18^ and Kibel et al.^19^ by STATA software version 12.0. Mantel-Haenszel fix effects model was used to estimate the CSM for the three studies for the I^2^ = 33.0% (P = 0.225). The pooled HR of CSM was (0.51 [95% CI 0.30–0.73], P<0.001) with a low heterogeneity (Figure 2).

All the studies were prospective or retrospective cohort studies, except for four RCTs^12^,^13^,^14^,^15^ and one case-controlled study^22^. The Jadad scale of each of the four RCTs was 3 points; therefore, the studies were considered to be high quality (Table 2). The NOS quality assessments of the 10 cohort studies showed satisfactory results, with star ratings of ≥7 (Table 3). The NOS score of the case-controlled study was 8^22^.

### Results of included studies

#### Studies without combined regimens (N = 6)

##### RP versus RT (N = 2)

Zelefsky et al. included 2380 patients with pathologically confirmed T1c-3b stage PCa, of whom 409 were high-risk^16^. Among the high-risk group, the 8-yr CSM was 3.8% for RP and 9.5% for RT (P = 0.015). The absolute difference (AD) between groups (using RP−RT) for 8-yr distal metastasis-free survival (DMFS) was higher in patients with high-risk tumors compared with those with intermediate-risk or low-risk tumors (high-risk, intermediate-risk, and low-risk: 7.8%, 3.3%, and 1.9%, respectively).

Merino et al. performed a retrospective cohort study^17^. Of the 1200 patients with clinically localized PCa, 294 patients were high-risk (216 in RP vs. 78 in RT). Stratified analysis revealed that RP resulted in a better outcome regarding 7-year OS (87.5% vs. 77.3%, *P* = 0.02); however, there was no significant difference between high-risk patients in CSS (85.4% for RT vs. 93.0% for RP, *P* = 0.07) and those in CSM (RT vs. RP: hazard ratio [HR] 1.71, *P* = 0.218).

##### RP versus RT versus WW (N = 1)

Tewari et al. included patients with high-risk localized PCa with Gleason 8–10^18^. Among these, 197 received WW, 137 received RT, and 119 underwent RP. The risk of CSM in patients who underwent RP was 49% and 68% lower than that in patients treated with RT and WW, respectively (HR 0.51, *P* = 0.053; HR 0.32, *P* = 0.001, respectively). The difference between RT and WW was also significant (HR 0.64, *P* = 0.018).

##### RP versus RT versus BT (N = 2)

Kibel et al. did a prospective combined retrospective study of 10,429 patients with localized PCa^19^, of whom 1201 were high-risk. Of the high-risk patients, 525 underwent RP, 676 received RT, and 33 received BT. Multivariate analysis revealed that the differences in OS were significant (RP vs. RT: HR 1.7, *P* = 0.001; RP vs. BT: HR 3.1, *P* < 0.001). However, there was no difference in CSM among the treatment groups (RT vs. RP: HR 1.3, *P* = 0.2; BT vs. RP: HR 1.6, *P* = 0.5). Unfortunately, the comparison of RT and BT groups was not available.

A study performed by Stokes et al. compared the long-term biochemical disease-free survival (BDFS) for patients undergoing RP, external beam radiotherapy (EBRT) and BT alone^20^. Of the 318 patients included, 268 were high-risk (RP vs. EBRT vs. BT: *N* = 134 vs. 95 vs. 39, respectively). There was no significant difference between BT and RT for 5-yr BDFS, although the former had a higher result. A significant improvement was observed with RP (RP vs. RT, RP vs. BT, and RT vs. BT: *P* < 0.0001, *P* = 0.0136, and *P* = 0.1928, respectively).

##### RP versus RT versus ADT (N = 1)

The CaPSURE study was a prospective and retrospective cohort study, with a large sample size of localized PCa patients (*N* = 8982)^21^. Of the 7538 patients whose data were available, 5066 underwent RP, 1143 received RT, and 1329 received ADT. The study made systemic predictions of the likelihood of OS, PFS, and pathological stage according to the CAPRA (California San Francisco Cancer of the Prostate Risk Assessment) scoring systems (Table 1). After stratification using the CAPRA, the differences in HR (using RT−RP, ADT−RT, or ADT−RP) for 10-yr CSM increased with higher CAPRA scores (Table 4). Data revealed that the 10-yr CSM of RP and RT were similar in moderate-risk and low-risk patients. However, the 10-yr CSM in high-risk patients treated using RP was significantly lower than that in high-risk patients treated using RT.

#### Studies with combined regimens (N = 12)

##### RP versus RP + ADT (N = 2)

Siddiqui et al. performed a case-controlled study, which enrolled 191 pT3bN0M0 patients who underwent adjuvant ADT (aADT) and then matched them (1:1) with a control group receiving RP alone^22^. Finallly, the RP + aADT group experienced improved 10-yr CSS (94% vs. 87%, *P* = 0.037). However, the 10-yr OS was similar between groups (75% vs. 69%, *P* = 0.12). A cohort study by Bastide et al. also compared the use of RP (*N* = 82) with RP plus aADT (*N* = 41) for pT3bN0M0 patients^23^. Multivariate analysis showed that the combined group had a lower PSA-biochemical recurrence rate (HR 0.64, *P* = 0.13).

##### RP + ADT versus RT + ADT (N = 1)

Koie et al. reviewed 329 high-risk localized PCa patients that were treated with RP plus nADT (*N* = 216) or RT plus nADT (*N* = 81)^24^. Propensity-score matching identified 78 matched pairs of patients with similar baseline data. Kaplan-Meier analysis showed that 3-yr OS was 98.3% and 92.1% in RP and RT groups, respectively (*P* = 0.156). There were also no significant differences in the 3-yr biochemical recurrence-free survival rates (BRFS) (RP vs. RT: 86.4% vs. 89.4%, *P* = 0.878).

##### RP versus RT+ADT (N = 2)

A recent study by Lee et al. included patients with high-risk localized PCa that was treated with RP (*N* = 251) or RT plus neoadjuvant ADT (nADT) (*N* = 125)^25^. Results showed that the 5-yr CSS was longer in patients treated with RP compared with the combined group (96.5% vs. 88.3%). Multivariate analysis showed that CSM increased significantly in the combined group than RP group (HR 3.22, *P* = 0.001). A similar study by Hsu et al. compared patients with T3a PCa, treated with RP (n = 200) or RT plus nADT (*N* = 35)^26^. The long-term survival outcomes were better for RP, although OS (95.9% vs. 79.8%, *P* = 0.21) and CSS (98.7% vs. 88.7%, *P* = 0.42) differences were not significant.

##### RP versus RT + BT + ADT (N = 1)

Westover et al. compared treatment using RP with EBRT + BT + (neoadjuvant + concurrent) ADT in patients with localized PCa with a Gleason score of 8–10^27^. Multivariate analysis showed that RP was not associated with an increased risk of CSM compared with the combination group (HR 1.8 [0.6–5.5], *P* = 0.3).

##### RT versus RT + ADT (N = 4)

A RCT conducted by Bolla et al. reported that RT plus 3-yr aADT resulted in a significantly better 5-yr OS than RT alone (79% for the combination vs. 62% for RT, *P* = 0.001)^12^. D'Amico et al. also performed a comparison between RT and RT plus 6-mo aADT^13^. Significant difference was also found for 5-yr OS (88% vs. 78%, *P* = 0.04). Pilepich et al. also reported a better CSS using RT plus aADT (63.5% vs. 48.2% *P* = 0.01) than RT alone^14^. Similarly, Miljenko, et al. revealed a better outcome using RT plus (n + c) ADT, although the difference was not significant (8-yr OS 38% vs. 31%, P = 0.98; 8-yr CSM 44% vs. 54%, P = 0.36)^15^.

##### RT + BT versus RT + BT + ADT (N = 2)

Galalae et al. included 611 patients with localized PCa^28^. Among these, 359 were at high-risk, and were treated with RT and high-dose rate BT (HDR-BT) either combined with (*N* = 119) or without (*N* = 240) (n + c) ADT. Multiple regression analyses showed that the “no ADT” group had a better outcome regarding OS (87% vs. 80%, *P* = 0.057) and CSS (97% vs. 90%, *P* = 0.002). Demanes et al. performed a similar study of 113 patients with high-risk PCa^29^, with 65 and 48 in the “ADT” group (RT + BT + ADT) and “no ADT” group, respectively. Kaplan-Meier analysis demonstrated improved 10-yr PSA-progression-free survival (PSA-PFS) in patients in “ADT” group, but not significantly (70% vs. 62%, *P* > 0.05).

The results of each study are shown in Tables 4.

## Discussion

Recent treatment options for high-risk PCa include RP, RT, BT, ADT, WW, and combined schemes^30^. However, the optimal first-line treatment for clinically high-risk PCa remains controversial^31^. Current guidelines are inconsistent between the EAU, AUA, and NCCN^32^,^33^,^34^. EAU and AUA preferred to choose RP as the first step for patients with high-risk PCa; EAU also suggested that ADT should be given to patients after RP. Conversely, the initial option recommended by NCCN was combined RT with ADT. Therefore, the current study focused on a comparison of all available approaches.

To our knowledge, this is the first systematic review comparing the long-term survival of patients with high-risk PCa treated with all available approaches (RP, RT, BT, ADT and WW, combined or alone). One of the most important outcomes of our systematic review of 18 longitudinal studies is that RP/(RT plus aADT) has the best survival outcome in patients with high-risk PCa. Conversely, WW had the worst outcome. The overall priority for treatment strategy could be ranked as follows: RP/(RT plus aADT), RT, and ADT/WW. RP has significant better OS and BDFS than RT or BT. RP can significantly decrease 49% of the CSM than RT alone based on the meta-analysis results. Although ADT can improve the CSS of RP, we do not recommend the regimens of (RP+aADT) because this conclusion is based on a case-control study^22^; when comparing RP with (RT+ADT), added ADT to RT still fail to challenge the position of RP for the latter has significant lower CSM; ADT can improve the OS and CSS of RT significantly, but the outcomes are inconsistent when ADT is added to regimens of (RT+ BT). In brief, RP/(RT + aADT) is the appropriate first-line therapy regimen for high-risk PCa. For patients who can tolerate surgery, RP is the preferred choice; RP plus aADT regimen can be used for clinical T3b patients. For patients who are vulnerable to comorbidities, such as the elderly, the alternative is (RT+aADT); the conclusions of (RT + BT) with/without ADT are not inconsistent.

It seemed that consensus had been reached on this issue after the current systematic review that RP had better survival outcomes, but it did not mean that RP possessed the overwhelming advantage than other approaches, especially than RT plus aADT. In fact, the treatment option was affected by many different factors, and thus it was unwise of urologists to select RP for all patients with high-risk PCa. For example, a 20-year follow-up study by Peter et al. analyzed 767 patients with localized PCa that were treated using observation or ADT^35^. Of the 138 deaths with a Gleason score of 8–10, 53% were due to PCa and 24% due to other causes (such as comorbidities including diabetes mellitus or hypertension). In addition, a cohort of localized PCa patients assessed by Lu-Yao et al. had a median age of 78 yr^36^, and was managed conservatively without surgery or radiation. After a median 8.3-yr follow-up, the 10-yr death rate for poorly differentiated subgroup was 25.6% due to PCa, and 56.5% due to other causes. These two studies revealed that a competing medical condition could affect survival outcomes, particularly in elder patients who were vulnerable to comorbidities. In contrast, the possibility of complications, such as incontinence and erectile dysfunction, should be taken into account in younger individuals who are candidates for RP. Ward et al. reported a 15-yr study assessing RP use in patients presenting locally advanced (cT3) PCa^37^. Among these, 21% suffered incontinence after 1 yr, and 75% had no erectile function after RP (only 26% with bilateral or unilateral nerve preservation). Therefore, although we had drew conclusion that RP had better survival outcomes, different treatment options should be considered according to the tumors, patient age, concomitant diseases, and individual preferences.

In studies that included combined regimens, ADT was used widely to improve the survival outcomes of RP, RT, or BT. ADT use before surgery or radiotherapy was based on “the first hypothesis” that androgen ablation might reduce the tumor bulk and enhance tumor cell kill to enable total excision of the cancerous area, whereas ADT use after surgery or radiotherapy might eliminate residual tumor cells in the primary lesions and subclinical metastatic lesions (called “the second hypothesis”). Studies by Siddiqui et al.^22^ and Bastide et al.^23^ demonstrated that nADT before RP could improve long-term survival outcomes. However, both these studies included only high-risk patients with seminal vesicle invasion (pT3b); therefore, we cannot reach the same conclusion for localized PCa patients with clinical stage ≥ T2c, Gleason 8–10, or PSA>20 ng/ml. In addition, four RCTs with the addition of aADT to RT^12^,^13^,^14^,^15^ substantially verified “the second hypothesis”. But for “the first hypothesis”, it was not safe to draw a positive conclusion because the inconsistency of Galalae et al. and Demanes et al.^28^,^29^. Specifically, better outcome in the “no ADT” group in the study by Galalae et al. was unexpected^28^. One explanation for this is the obvious selection bias in the “ADT” group because ADT therapy was performed specially in patients with an enlarged prostate. An alternative explanation is that patients did not benefit from the short duration of ADT. Therefore, additional high-quality RCTs are needed to establish the value of nADT before RT plus BT for high-risk PCa.

Although the 14 large-scale longitudinal studies included in this systematic review provided evidence to allow robust conclusions to be drawn, the review has several limitations. First, many studies had limited methodological quality. The definition of high-risk PCa, RT doses and cycles, start time of follow-up and median follow-up durations varied among studies. This also made it no value to conduct a formal meta-analysis for most of the included studies and the available meta-analysis was conducted only based on three studies. Second, RCTs that compared the long-term survival of RP and other approaches directly in patients with high-risk PCa are still unavailable; most of the available studies included patients with localized PCa; therefore, high-risk patients were discussed as a subgroup. Much baseline data (e.g. mean age and mean PSA) were not reported, making comparisons among studies challenging. Finally, important issues such as perioperative complications and cost effectiveness of RP and RT were not assessed. Treatment costs should be considered, particularly in developing countries such as China. Therefore, further large scale, rigorous RCTs with consistent inclusion criteria, design and outcome measures are strongly desirable to ascertain the long-term outcomes, safety, and cost-effectiveness of the different treatment approaches.

In summary, this systematic review provided strong evidence to support RP or RT plus adjuvant ADT as first-line therapy option for high-risk PCa. Although RP resulted in the best survival outcomes, it was not suitable for all patients. In patients who could tolerate surgery, RP is the best choice, whereas the alternative is RT plus aADT in patients who are vulnerable to comorbidities. Urologists should fully discuss all treatment options with the patient and specialists from other related disciplines, and comprehensively consider various factors including the tumors and patient preferences. This would allow treatment benefits to be expanded to their fullest potential in all patients.

## Methods

### Search strategy

We searched for relevant studies (search date, July 12, 2014) using OvidSP to search three databases: Ovid MEDLINE® (1946 to present), EMBASE® (1974 to July 19, 2014), and the Cochrane Central Register of Controlled Trials® (June 2014). The search strategy was as follows: [(Prostatic Neoplasms or prostate cancer).sh. or (Prostate Neoplasm or carcinoma of prostate).tw.] and [(high-risk or High-grade).tw.] and [(prostatectomy or Radiation or Radiotherapy or Brachytherapy or watchful waiting or observation).sh. or (radical prostatectomy or radiation therapy or androgen-deprivation therapy or seed implantation or active surveillance).tw.]. The meaning of “sh” and “tw” were MeSH heading and text word, respectively. The reference lists of the included studies, other reviews, and related articles not identified by our electronic searches were also screened for additional possible studies. Our literatures search had no language and publication status restrictions. The gender was limited to male. Two authors (Lin and Cao) then reviewed the titles, abstracts, and the full text of each article, independently. Any disagreements were solved by discussion within the study group.

### Study selection

Studies that met all of the following criteria were included: longitudinal studies a) in which the study population or subpopulation included high-risk PCa patients, b) using RP, RT, BT, ADT, or WW as the study variables or exposure variables and c) that reported quantitative end-points comparing the effect between or among RP, RT, BT, ADT, or WW [e.g., OS, CSS, and cancer-specific mortality (CSM)] with at least three years' median follow-up. High-risk PCa was defined as clinical stage ≥T2c, Gleason score 8–10, or PSA > 20 ng/ml, with a negative computerized tomography or bone scan.

### Data extraction

Data from the included studies were extracted by two reviewers (J.H. and D.H.) and cross-checked, respectively. Any disagreements were reconciled by a third person (L.R. or Q.W.). The following information was collected from the reports of original trials: first author, study design, sites, time, population, median follow-up, comparison of treatment, end-points and definition of high-risk PCa. Discrepancies were resolved in consulation with Q.W.

### Quality assessment

The methodological quality of the included studies was evaluated according to the Jadad scale for randomized controlled trials (RCTs)^9^ and the Newcastle-Ottawa Scale (NOS) using a “star system” for cohort or case-controlled studies^10^,^11^. Scores ≥3 points and ≥7 points were considered high quality using the Jadad scale and NOS, respectively.

### Statistical analysis

The log hazard ratio (HR) was chosen as the appropriate summary statistics because it was the only summary statistic that allows for both censoring and time to an event. Possible heterogeneity of studies was quantified using the chi-squared test and I^2^ value. If I^2^ < 50% or P value > 0.10 (considerable lower heterogeneity), the fixed effect model was chosen; otherwise the random effect model was used. An observed HR > 1 indicated a worse outcome for the positive group compared to the negative group and was considered significant if the 95% CI did not overlap 1. We followed the PRISMA statement for conducting a high-quality meta-analysis. All analyses were performed using STATA software version 12.0 (STATA Corporation, College Station, USA) analysis.

## Figures and Tables

**Table 1:** Characteristics of included studies (*N* = 18)

Study ID	study design	Sites/Time	Population/Median age(RP vs. RT or Alone vs. combination)	Median follow-up	Comparison of treatment/N	End-points	Definition of high-risk PCa
**Studies without combined regimens (*N* = 6)**							
Zelefsky et al. 2010 [16]	R. cohort	New York, US 1993-2002	Clinical stages T1c-T3b/60(55–65) vs. 69(64–73)	RP: 5 yr EBRT: 5.1 yr	RP vs. EBRT 1318 vs. 1062	CSM, DMFS	T3, Gleason 8–10, or PSA>20ng/ml
Merino et al. 2013 [17]	R. cohort	Chile 1990–2010	Localized PCa/63 (62.6–63.5) vs. 70(69–71)	RP: 91.7 mo IMRT: 76 mo	RP vs. IMRT 993 vs. 207	OS, CSS, BDFS	D'Amico definition
Tewari et al. 2007 [18]	R. cohort	New York, US 1980–1997	High-risk localized PCa 62.9 ± 6.2 vs. 68.1 ± 5.8	RP vs. EBRT vs. WW 68 vs. 53 vs. 52 mo	RP vs. EBRT vs. WW 119 vs. 137 vs. 197	OS,CSS	Poorly differentiated, Gleason 8–10
Kibel et al. 2012 [19]	P. and R. cohort	Missouri/Ohio, US 1995–2005	Localized PCa 60(56–65) vs. 69(63–73)	67 mo	RP vs. EBRT vs. BT 6485vs. 2264 vs.1680	OS, CSM	D'Amico definition
Stokes et al. 2000 [20]	R. cohort	Alabama, US 1988–2000	Localized PCa 66(43–79) vs. 72(49–87)	RP vs. EBRT vs. SI 66 vs. 72 vs.74 mo	RP vs. EBRT vs. SI 222 vs. 132 vs. 186	BDFS	T2c, T3, Gleason 7–10, or PSA>20 ng/ml
Cooperberg et al. 2010 [21]	P. and R. cohort	40 community-based centers, US 1995–2008	Localized PCa 62(56–67) vs. 72(67–75)	NA	RP vs. EBRT vs. ADT 5066 vs. 1143 vs. 1329	CSM, ACM	CAPRA Score 6~10
**Studies with combined regimens(*N* = 12)**							
Siddiqui et al. 2011 [22]	Case-controlled study	Rochester, US 1987–2010	Localized T3b PCa/66(48–78) vs. 65(48–79)	10 yr	RP vs. RP+aADT 191 vs.191	OS, CSS	No
Bastide et al. 2011 [23]	R. cohort	Aixen, France 1994–2008	Localized T3b PCa/65.3 vs. 63.7	60.3 mo	RP vs. RP+aADT 82 vs. 41	BCR	No
Koie et al. 2014 [24]	R. cohort	Hirosaki, Japan 2004–2012	High-risk localized PCa/71 vs. 73.5	RP+nADTvs.RT+nADT 37.6 vs.31.5 mo	RP+nADT vs. RT+nADT 216 vs. 81	OS, BRFS	T2c/T3, Gleason 8–10,PSA>20 ng/ml
Lee et al. 2014 [25]	R. cohort	Seoul, Korea 1990–2009	High-risk localized PCa/67.5 ± 7.0 vs. 68.6 ± 8.4	76 mo	RP vs. EBRT+(n+a)ADT 251 vs. 125	CSM	NCCN definition
Hsu et al. 2006 [26]	R. cohort	Leuven, Belgium 1987–2004	Unilateral cT3 PCa/63.3(41–79) vs.65.1(51–75)	74.7 mo	RP vs. RT+nADT 200 vs. 35	OS, CSS	No
Westover et al. 2012 [27]	R. cohort	Boston/Durham, US 1988–2009	Localized PCa, and Gleason 8–10/65(58–69) vs.70(66–73)	4.62 yr	RP vs. RT+BT+(n+c)ADT 285 vs. 372	CSM	D'Amico definition
Bolla et al. 1997 [12]	RCT	France, Netherlands, Switzerland1987–1995	High-grade localized and locally advanced PCa/70(51–80) vs. 71(54–80)	45 mo	RT vs. RT+(c+a)ADT 198 vs. 203	OS	No
D'Amico et al. 2004 [13]	RCT	Harvard outreach, US 1995–2001	Intermediate and high-risk localized PCa/73(51–81) vs. 72(49–82)	4.52 yr	CRT vs. CRT+(c+a)ADT 104 vs. 102	Time to PSA failure, OS	Gleason 7–10, PSA>20 ng/ml
Pilepich et al. 1997 [14]	RCT	Scranton/Wisconsin,US, 1987–1992	PCa with cT3 or regional node involved/	4.5 yr	RT vs. RT+aADT 468 vs. 477	CSS	Gleason 8–10
Pilepich et al. 2001 [15]	RCT	California/Sacramento, US, 1987–1991	Bulky PCa (T2–T4) with/without pelvic node involvement/	6.7 yr	RT vs. RT+(n+c)ADT 230 vs. 226	OS, CSM	Gleason 8–10
Galalae et al. 2004 [28]	P. cohort	Germany; US 1986–2000	Localized PCa/	5 yr	HDR-BT+ EBRT vs. HDR-BT +EBRT + (n+c)ADT: 434 vs. 177	OS,CSS	NCCN definition
Demanes et al. 2009 [29]	P. cohort	Oakland, CA1991–2008	Localized PCa/	6.4 yr	BT+EBRT vs. BT+EBRT+nADT 211 vs. 200	OS, BC	NCCN definition

P. and R. Cohort: prospective and retrospective cohort study. RCT: randomized controlled trial. EBRT: external beam radiotherapy. CRT: 3-dimensional conformal radiation therapy. ADT: androgen-deprivation therapy. nADT: neoadjuvant ADT. (n+a)ADT: neoadjuvant and adjuvant ADT. (n+c)ADT: neoadjuvant and concurrent ADT. IMRT: intensity-modulated radiation therapy. BT: brachytherapy. HDR-BT: High Dose Rate BT represented one form of BT. SI: Seed implantation represented the other form of BT. WW: watchful waiting. OS: Overall Survival. CSS: Cancer-Specific Survival. CSM: Cancer-Specific Mortality. ACM: All-Cause Mortality (To assess perioperative mortality and death from complicationsof radiation). DMFS: Distant Metastases-Free Survival. BDFS: Biochemical Disease-Free Survival. BRFS: biochemical recurrence-free survival. BC: Biochemical control of PSA testing. BCR: Biochemical recurrence of PSA testing. D'Amico definition: PSA >20ng/ml, Gleason score 8–10, or clinical stage ≥ T2c. NCCN definition: PSA >20ng/ml, Gleason score 8–10, or clinical stage ≥T3a. CAPRA Score: Ranging from 0–10, mainly based on PSA levels, biopsy Gleason grade, clinical T stage, age at diagnosis and percentage of positive biopsy cores; a score of 6–10 points was considered to be high-risk. Dates were not available. NA: not applicable.

**Table 2:** Quality assessments of RCTs with Jadad Score (*N* = 4)

Items	Score Standard			Study ID			
	0	1	2	Bolla et al. 1997 [12]	D'Amico et al.2004 [13]	Pilepich et al.1997 [14]	Miljenko, et al. 2001 [15]
Randomization	Not randomized or inappropriate method of randomization.	The study was described as randomized.	The method of randomization was described appropriately.	2	2	2	2
Double blinding	No blind or inappropriate method of blinding.	The study was described as double blind.	The method of double blinding was described appropriately.	0	0	0	0
Withdrawals and dropouts	Not describe the follow-up.	A description of withdrawals and dropouts.	/	1	1	1	1
Score summaries	/	/	/	3	3	3	3

The full mark for Jadad Score was 5-point. Scores ≥3 was considered with high-quality.

**Table 3:** Quality assessments of cohort studies with Newcastle-Ottawa Scale (NOS) (*N* = 13)

Study ID	Representativeness of the exposed cohort	Selection of the non exposed cohort	Ascertainment of exposure	Demonstration that outcome of interest was not present at baseline	Comparability of cohorts on the basis of the design or analysis	Assessment of outcome	Was follow-up long enough for outcomes' occur	Adequacy of follow up of cohorts	NOS quality score (Num. of stars)
Zelefsky et al. 2010 [16]	⋆	⋆	⋆	⋆	⋆	⋆	⋆	⋆	8
Merino et al. 2013 [17]	⋆	⋆	⋆	⋆	NA	⋆	⋆	⋆	7
Tewari et al. 2007 [18]	⋆	⋆	⋆	⋆	⋆⋆	⋆	⋆	⋆	9
Kibel et al. 2012 [19]	⋆	⋆	⋆	⋆	NA	⋆	⋆	⋆	7
Stokes et al. 2000 [20]	⋆	⋆	⋆	⋆	NA	⋆	⋆	⋆	7
Cooperberg et al. 2010 [21]	⋆	⋆	⋆	⋆	⋆	⋆	⋆	⋆	8
Bastide et al. 2011 [23]	⋆	⋆	⋆	⋆	⋆⋆	⋆	⋆	⋆	9
Koie et al. 2014 [24]	⋆	⋆	⋆	⋆	⋆⋆	⋆	⋆	⋆	9
Lee et al. 2014 [25]	⋆	⋆	⋆	⋆	⋆⋆	⋆	⋆	⋆	9
Hsu et al. 2006 [26]	⋆	⋆	⋆	⋆	⋆	⋆	⋆	⋆	8
Westover et al. 2012 [27]	⋆	⋆	⋆	⋆	⋆⋆	⋆	⋆	⋆	9
Galalae et al. 2004 [28]	⋆	⋆	⋆	⋆	NA	⋆	⋆	⋆	7
Demanes et al. 2009 [29]	⋆	⋆	⋆	⋆	NA	⋆	⋆	⋆	7

The full mark for NOS was 9-point. Scores ≥7 was considered with high-quality. NA: Not Available.

**Table 4:** Results of high-risk group/subgroup of included studies (*N* = 18)

Study ID	Comparison of therapy/Simple size for high-risk group	RT/ADT regimen	Death counts for high-risk group due to PCa 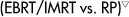	End-points			

				OS	CSS	CSM	Other end-points	
**Studies without combined regimens (*N* = 6)**							
Zelefsky et al. 2010 [16]	RP vs. EBRT 348 vs. 61	81 Gy or 86.4 Gy	13 vs. 6	NA	NA	RP > RT	AD of 8-y DMFS^a^: RP > RT
Merino et al. 2013 [17]	RP vs. IMRT 216 vs. 78	76 Gy	27 vs. 18	RP ≫RT§	RP > RT$	RP > RT	
Tewari et al. 2007 [18]	RP vs. EBRT vs. WW 119 vs. 137 vs. 197			NA	NA	RP > RT ≫ WW	
Kibel et al. 2012 [19]	RP vs. EBRT vs. BT 525 vs. 676 vs. 33	median dose: 7,400 cGy		RP ≫RT; RP ≫ BT	NA	RP ≫RT; RP > BT	
Stokes et al. 2000 [20]	RP vs. EBRT vs. SI 134 vs. 95 vs. 39	pelvis/periprostatic region: 4500 cGy/6500–7000 cGy		NA	NA	NA	5-y BDFS^b^: RP ≫RT > BT
Cooperberg et al. 2010 [21]	RP vs. EBRT vs. ADT 328 vs. 279 vs. 417			NA	NA	NA	D of HR for CSM^c^: RP > RT > ADT
**Studies with combined regimens (*N* = 12)**							
Siddiqui et al. 2011 [22]	RP vs. RP+aADT 191 vs.191			RP+aADT > RP	RP+aADT ≫ RP	NA	
Bastide et al. 2011 [23]	RP vs. RP+aADT82 vs. 41	ADT: 15 mo		NA	NA	NA	HR for PSA-BCR^d^: RP+aADT > RP
Koie et al. 2014 [24]	RP+nADT vs. RT+nADT 78 vs. 78	RT: 70–76 Gy ADT: 6 mo		RP+nADT > RT+nADT	NA	NA	3-y BDFS: RT+nADT > RP+nADT
Lee et al. 2014 [25]	RP vs. RT+(n+a)ADT 251 vs. 125	RT: 6–10 MV, 74–79 Gy	RP vs.EBRT+(n+a)ADT 12 vs. 23	NA	RP > RT+(n+a)ADT	RP ≫ RT+(n+a)ADT	
Hsu et al. 2006 [26]	RP vs. RT+nADT 200 vs. 35	nADT: varied cross patients	RP vs. RT+nADT 8 vs. 7	RP > RT+nADT	RP > RT+nADT	NA	
Westover et al. 2012 [27]	RP vs. Combination 285 vs. 372	RT: 45 Gy; ADT: 4.3 mo BT: ^125 ^I or ^103^ Pd	RP vs. Combination 15 vs. 6	NA	NA	RT+BT+(n+c)ADT>RP	
Bolla et al. 1997 [12]	RT vs. RT+(c+a)ADT 198 vs. 203	RT: 70 Gy; ADT: cyproterone acetate, goserelin	RT+aADT vs. RT 6 vs. 26	RT+aADT ≫ RT	NA	NA	
D'Amico et al. 2004 [13]	RT vs. RT+(c+a)ADT& 77 vs. 76	RT:70.35 Gy; ADT: leuprolide acetate/goserelin,6 mo		RT+aADT ≫ RT	NA	NA	
Pilepich et al. 1997 [14]	RT vs. RT+aADT 110 vs. 115	RT: 65–70 Gy; ADT: goserelin 3.6mg	RT vs. RT+aADT40 vs. 25	NA	RT+aADT ≫ RT	NA	
Pilepich et al. 2001 [15]	RT vs. RT+(n+c)ADT 67 vs. 57	RT: 65–70 Gy; ADT: Goserelin/Flutamide 3.6mg/250 mg	RT vs. RT+(n+c)ADT 46 vs. 37	RT+(n+c)ADT > RT	NA	RT+(n+c)ADT > RT	
G 2004 [28]	BT+RT vs. “ADT”^e ^240 vs. 119	BT: iridium-192, 370 GBq; EBRT:45.6–50 Gy; ADT: 4 mo	“No ADT” vs. “ADT”^f ^7 vs. 12	“No ADT” > “ADT”	“No ADT” ≫ “ADT”	NA	
Demanes et al. 2009 [29]	BT+RT vs. BT+RT+nADT 48 vs. 65	BT: 5.5–6.0 Gy; RT: 36.0–39.6 Gy ADT: 4.6 mo		NA	NA	NA	10-y PSA-PFS^g^ (No-ADT vs. ADT): 62%vs.70%,p>0.05

§A≫B: A had better survival outcome than B for the corresponding end-point, and the difference between A and B was significant.

$A>B: A had better survival outcome than B for the corresponding end-point, but the difference between A and B was non-significant.

&The outcome of OS of the study was applied for the intermediate and high-risk localized PCa; the subgroup analysis for high-risk PCa was not conducted actually.

a. AD of 8-y DMFS: The Absolute Difference (AD) between groups (using RP-RT) for 8-year Distal Metastases-Free Survival.

b. BDFS: Biochemical Disease-Free Survival.

c. The differences of HR for 10-year CSM: 0/2/4/5/6/8/10 point (6–10 points: high-risk): (using RT-RP) 0.62/1.19/2.23/3.03/4.07/6.94/10.41; (ADT-RT) 1.19/2.07/4.22/5.64/7.42/11.81/15.57; (ADT-RP) 1.81/3.54/6.45/8.67/11.49/18.75/25.98.

d. PSA-BCR: Biochemical recurrence of PSA testing.

e. “ADT” means RT+BT+(n+c)ADT.

f. “No ADT” vs. “ADT” = BT+EBRT vs. BT+EBRT+ADT.

g. PSA-progression-free survival. Dates were not available. NA: Not applicable.
